# Coagulation factor 9-deficient mice are protected against dextran sulfate sodium-induced colitis

**DOI:** 10.1242/bio.034140

**Published:** 2018-06-26

**Authors:** Avinash Khandagale, Jens M. Kittner, Amrit Mann, Stefanie Ascher, Bettina Kollar, Christoph Reinhardt

**Affiliations:** 1 Center for Thrombosis and Hemostasis (CTH), University Medical Center Mainz, Johannes Gutenberg University Mainz, Langenbeckstrasse 1, 55131 Mainz, Germany; 2 Institute of Environmental Medicine, Karolinska Institutet, SE-171 77 Stockholm, Sweden; 3 I. Department of Medicine, University Medical Center Mainz, Johannes Gutenberg University Mainz, Langenbeckstrasse 1, 55131 Mainz, Germany; 4 German Center for Cardiovascular Research (DZHK), Partner Site RheinMain, Mainz, Germany

**Keywords:** Hemophilia B, Coagulation factor IX, Toll-like receptor-2, Colitis, Microbiota

## Abstract

Patients with inflammatory bowel disease (IBD) are susceptible to thromboembolism. Interestingly, IBD occurs less frequently in patients with inherited bleeding disorders. Therefore, we analyzed whether *F9*-deficiency is protective against the onset of acute colitis in a genetic hemophilia B mouse model. In the 3.5% dextran sulfate sodium (DSS)-induced colitis model, *F9*-deficient mice were protected from body-weight loss and had a reduced disease activity score. We detected decreased colonic myeloperoxidase activity and decreased CXCL1 levels in DSS-treated *F9*-deficient mice compared with wild-type (WT) littermate controls, indicating decreased neutrophil infiltration. Remarkably, we identified expression of coagulation factor IX (FIX) protein in small intestinal epithelial cells (MODE-K). In epithelial cell cultures, cellular FIX protein expression was increased following stimulation with the bacterial Toll-like receptor agonists lipopolysaccharide, macrophage-activating lipopeptide-2 and Pam3CSK4. Thus, we revealed a protective role of *F9*-deficiency in DSS-induced colitis and identified the intestinal epithelium as a site of ectopic FIX.

This article has an associated First Person interview with the first author of the paper.

## Abstract (precis)

**Summary:** Since IBD incidence is less frequent in patients with inherited bleeding disorders, we explored and demonstrated that F9-deficiency is protective against DSS-induced acute colitis in a hemophilia B mouse model.

## INTRODUCTION

Inflammatory bowel diseases (IBD) including ulcerative colitis (UC) and Crohn's disease (CD) is a group of chronic, relapsing, immunological, inflammatory disorders of the gastrointestinal tract (Xavier and Podolsky, 2007). Microbiota-triggered Toll-like receptor (TLR) signaling is protective in mouse models of inflammatory bowel disease (IBD), but it is incompletely understood how gut microbiota-derived TLR ligands regulate tissue protective factors of the intestinal epithelium (Rakoff-Nahoum et al., 2004). CD and UC are characterized by a dysbiotic gut microbiota (Frank et al., 2007). Gut microbes control the development of IBD in the susceptible host (Xavier and Podolsky, 2007). Interestingly, in genetic mouse models with fecal transplantation of a colitogenic microbiota, the IBD phenotype is transferrable into wild-type (WT) mice (Garrett et al., 2010). Meanwhile, there are results from first clinical trials, testing fecal microbiota transplantation as a new therapeutic option in IBD (Mattner et al., 2016). Nevertheless, the pathogenesis of IBD remains poorly understood.

Interestingly, IBDs occur less frequently than expected in patients with inherited bleeding disorders (Thompson et al., 1995), but it is currently unknown whether ectopically synthetized coagulation factors in the intestine could be involved. Factor IX plasma levels were found to be increased during IBD (Alkim et al., 2011). Deficiency of coagulation factor IX (FIX) is the cause of hemophilia B, a severe X-linked inherited bleeding disorder with a prevalence of 1:25,000 (Schulman and Smith, 1952; Rogers and Herzog, 2015). Factor IX is efficiently activated via the binary complex of tissue factor with activated coagulation factor VII (FVIIa) (Østerud and Rapaport, 1977) and via the thrombin amplification loop by activated FXI (FXIa) (Gailani and Broze, 1991). Activated FIX (FIXa) is a component of the Xase complex (Ca^2+^/FVIIIa/FIXa), a key entity of the coagulation pathway. The Xase complex promotes the cleavage of FX into activated FXa and thus the generation of thrombin (Rogers and Herzog, 2015). The generation of thrombin activity in the plasma of hemophilia B patients is clearly dependent on FIX concentrations under stimulation conditions with low tissue factor concentrations and, therefore, this pathway was suggested to be relevant for pathophysiological thrombin generation (Xi et al., 1989). To date, it is unresolved whether the deficiency of FIX protects from the onset of colitis.

One pathway of how the coagulation system contributes to acute intestinal mucositis is through activation of protease-activated receptor (PAR) signaling (Borensztajn et al., 2009). This could be triggered by epithelial sources of tissue factor (Luther et al., 1996) and increased ectopic synthesis of clotting factors by the intestinal epithelium (Yamada and Nagai, 1996). Enhanced coagulation factor signaling via PAR1 was shown to depend on the commensal gut microbiota (Reinhardt et al., 2012) and this signaling route has been implicated in the pathogenesis of IBD (Danese et al., 2007). In Crohn's disease, hypercoagulability and platelet abnormalities with a threefold higher risk for development of systemic thrombosis were described (Sevenchenkova et al., 2015). During active IBD, blood coagulation is activated due to tissue factor synthesis in monocytes (Edwards et al., 1987) and tissue factor-dependent coagulation activation was related to disease activity (Anthoni et al., 2007).

Therefore, we hypothesized that FIXa-dependent FXa generation could aggravate the symptoms of IBD (Danese et al., 2007). It is unknown whether FIX as a component of the Xase complex is present in intestinal epithelial cells and if microbial-associated molecular patterns (MAMPs) can induce the ectopic expression of FIX. Here, we reveal in a dextran sulfate sodium (DSS) mouse model of acute intestinal inflammation, that *F9*-deficiency is protective against colitis. Furthermore, we demonstrate with a mouse-specific intestinal epithelial cell culture model that MAMP-stimulation augments the ectopic intestinal synthesis of FIX.

## RESULTS

### *F9*-deficiency protects against DSS-induced colitis

**Fig. 1.**
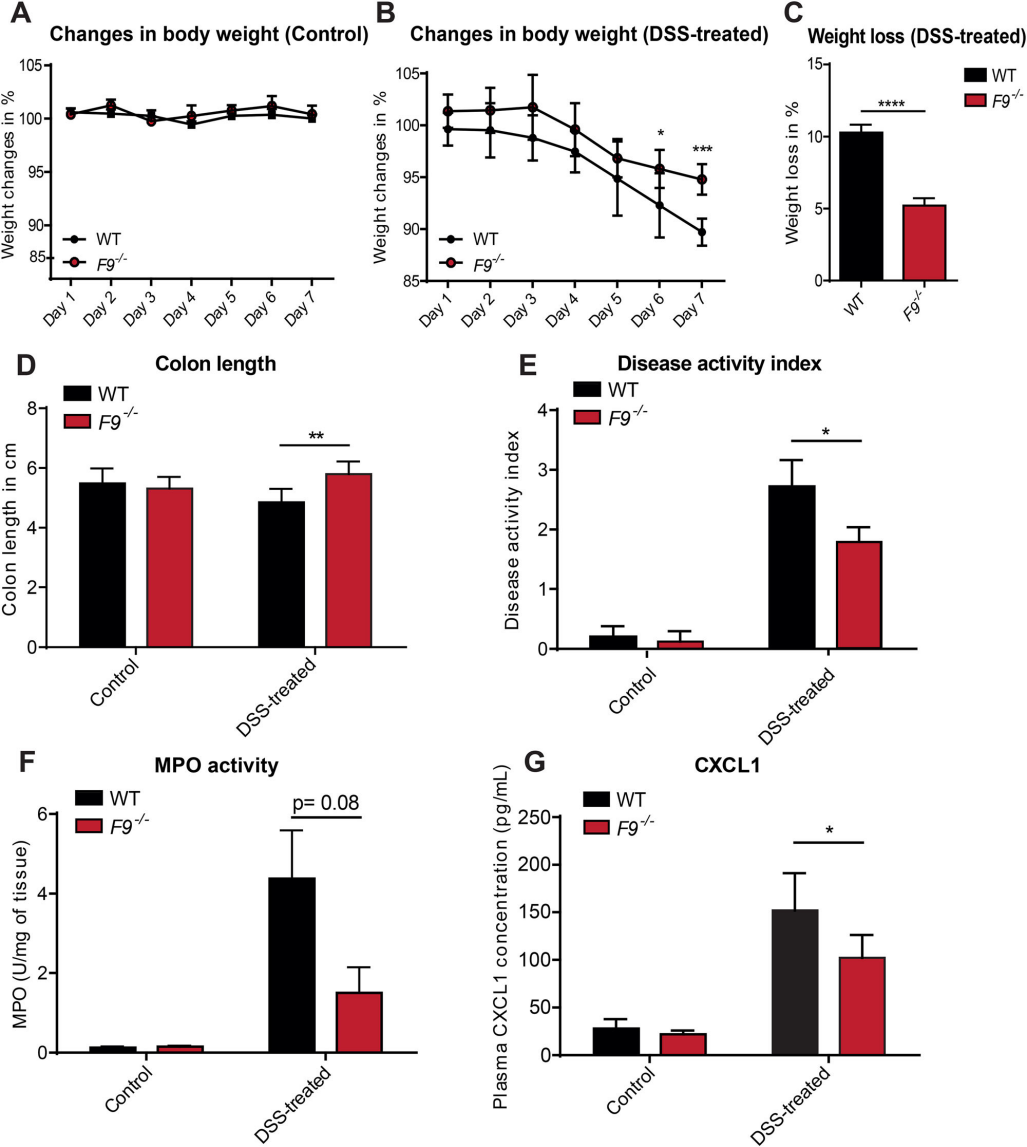
***F9^−/−^* mice are protected during DSS-induced colitis.** WT (*F9^+/+^*) and *F9^−/−^* mice were treated with 3.5% DSS in their drinking water for 7 days. (A) Mice were weighed and compared to their day 0 weights set as 100% (untreated control); (*n*=5–9 mice). (B) Weight loss was measured daily during treatment and is expressed as the average percentage of initial body weight±s.d; (*n*=6–8 mice). (C) Weight loss of the DSS-treated mice at day 7; average percentage of initial body weight±s.d; (*n*=6–8 mice). (D) Control mice (untreated) (*n*=5–9) or DSS-treated mice (*n*=6–8) were euthanized on day 7, and colons were removed and measured for their length. Results are expressed as the mean colon length±s.d. (E) Disease activity index was scored daily during treatment. The median score is reported for each group of mice: control, *n*=5–9; treated group, *n*=6–8 mice per group. (F) Colonic myeloperoxidase (MPO) activity in WT and *F9^−/−^* mice without (control) and with DSS administration was measured using absorbance values measured at 450 nm, *n*=4 mice per group. (G) Plasma CXCL1 levels were determined in plasma samples of DSS-treated (*n*=4–5 mice) and untreated WT and *F9^−/−^* mice (*n*=5–8 mice). *P<0.05; **P<0.01; ***P<0.001; ****P<0.0001.

As the prevalence of IBD was found to be reduced in hemophilic patients (Thompson et al., 1995), we were curious to explore whether coagulation factor IX-deficient (*F9^−/−^*) C57BL/6 mice (Lin et al., 1997) are protected against 3.5% DSS-induced acute intestinal inflammation. There was no difference in body-weight loss in the untreated control groups (Fig. 1A), but strikingly, the body-weight loss upon 3.5% DSS administration was significantly less in *F9^−/−^* mice compared with WT littermate controls (Fig. 1B,C). The reduced inflammation in DSS-treated *F9^−/−^* mice was further corroborated by significantly longer colons and a reduced disease activity index compared with DSS-treated WT controls (Fig. 1D,E). These parameters did not differ in untreated mice (control).

To assess the degree of infiltration with neutrophils, we analyzed the small intestinal and colon tissues of the DSS-treated mice. Reduced myeloperoxidase (MPO) activity indicated decreased neutrophil infiltration in the colon of *F9^−/−^* mice compared with DSS-treated WT controls (Fig. 1F**)**. MPO activity was also reduced in the small intestine, but not in the liver and the spleen (data not shown). Decreased neutrophil infiltration in *F9^−/−^* mice was also reflected by reduced plasma CXCL1 levels, a chemokine promoting neutrophil recruitment (Fig. 1G). Collectively, our results demonstrate that *F9*-deficiency protects against DSS-induced colitis.

### FIX protein levels in small intestinal epithelial cells are increased by stimulation with TLR agonists

**Fig. 2.**
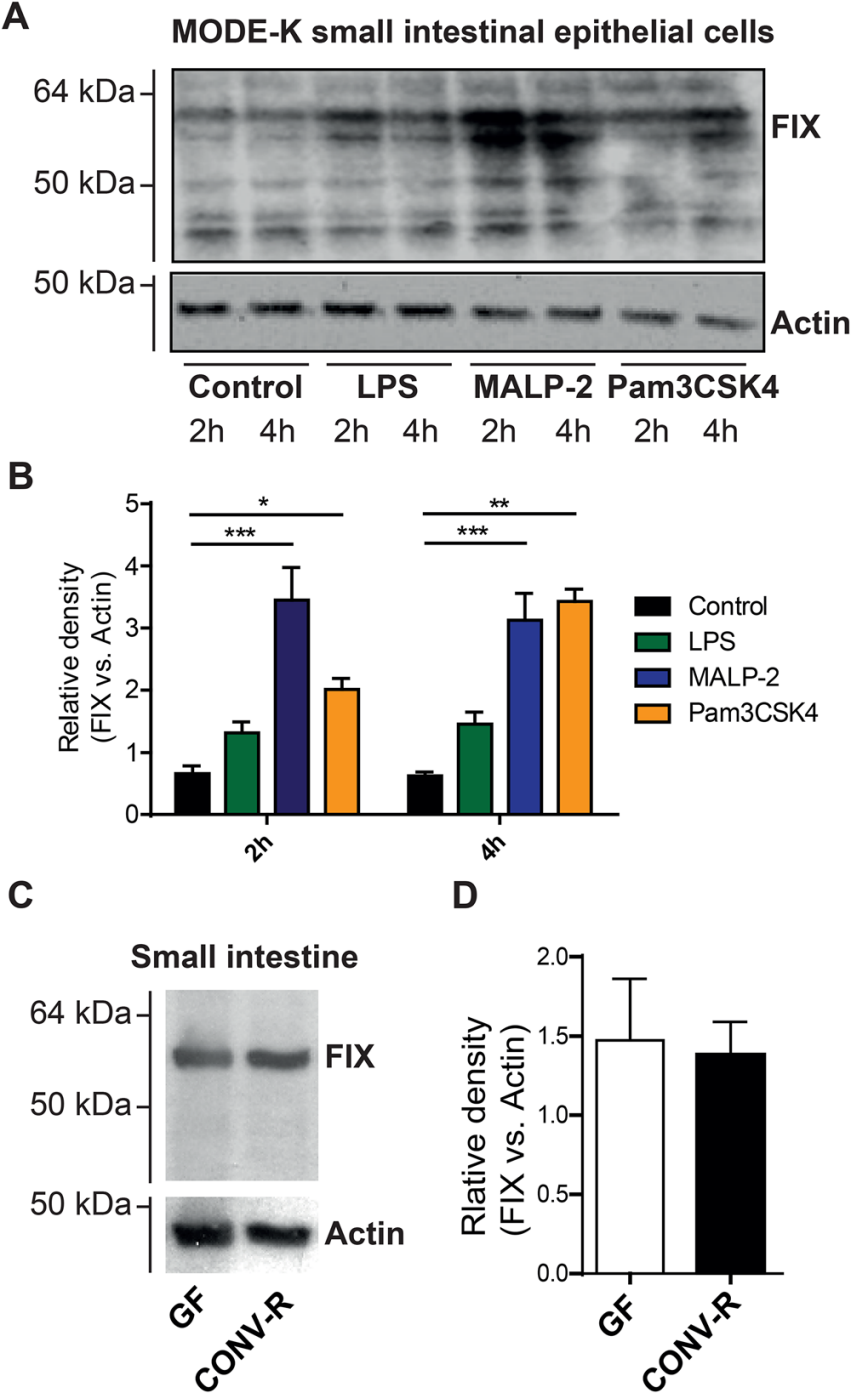
**Intestinal epithelial cell line responds with increased FIX expression to TLR activation.** (A) FIX immunoblot of vehicle treated or LPS (100 ng/ml), MALP-2 (2 µg/ml) or Pam3CSK4 (500 ng/ml) stimulated MODE-K cells for 2 and 4 h (*n*=3, representative blot). (B) Quantitative FIX band density with respect to the actin loading control. (C) FIX immunoblot analysis of small intestinal lysates of germ-free (GF) or conventionally-raised (CONV-R) mice (representative immunoblot, *n*=6–7 mice). (D) Corresponding FIX band density relative to the loading control actin band density. *P<0.05; **P<0.01; ***P<0.001.

To investigate whether activation of TLRs can trigger FIX protein expression in intestinal epithelial cells, we stimulated the immortalized mouse intestinal epithelial cell line MODE-K (Vidal et al., 1993) with the TLR4 agonist lipopolysaccharide (LPS), the TLR2/6 agonist macrophage-activating lipopeptide-2 (MALP-2), or the synthetic TLR2/1 agonist Pam3CSK4 for 2 and 4 h, respectively. Immunoblot analyses showed that TLR2 stimulation, in particular, increased epithelial FIX protein levels compared to unstimulated controls, but also LPS increased epithelial FIX levels to some extent (Fig. 2A,B). Our results demonstrate that TLR activation triggers ectopic FIX expression in small intestinal epithelial cells.

Next, we investigated whether FIX protein is differentially expressed in the distal small intestine of germ-free (GF) compared to conventionally-raised (CONV-R) Swiss Webster mice. Immunoblot analyses of small intestinal tissue lysates and densitometric analysis showed no differences in total small intestinal FIX protein levels (Fig. 2C,D). Thus, the gut microbiota did not affect ectopic FIX synthesis in the small intestine, suggesting that other mechanisms may be involved.

## DISCUSSION

Here, we used a *F9*-defecient hemophilia B mouse model to induce acute colitis using DSS to gain insights into the mechanism by which the coagulation pathway drives the inflammatory response to experimental colitis. In support of epidemiologic data (Thompson et al., 1995), our results demonstrated that the deficiency of FIX reduced the extent of inflammation in DSS-induced colitis. To our knowledge, we are the first to show that the anti-hemophilic clotting factor IX is present in intestinal epithelial cells and *F9*-deficiency rendered protection against colitis. We could show that TLR activation augments FIX protein levels in murine intestinal epithelial cells. FIX could be detected in small intestinal lysates, but its small intestinal expression levels were not influenced by the gut microbiota.

Our study demonstrated ectopic expression of the zymogen FIX in small intestinal epithelial cells. The finding that stimulation with TLR2 and TLR4 agonists increased FIX protein levels in the MODE-K cell culture model adds to the concept that the coagulation system has evolved as a host defense system to prevent invasion and spreading of microbes from the environment (Levin and Bang, 1964; Massberg et al., 2010). Our finding on the presence of FIX in the intestinal epithelium suggests that microbial patterns might augment clotting reactions at inflammatory epithelial interfaces.

In case of DSS-induced colitis, *F9*-deficiency reduces the extent of the acute intestinal inflammation. In the 3.5% DSS-colitis mouse model, *F9*-deficiency protected from weight loss and reduced colon length. Moreover, the deficiency of this clotting factor diminished the disease activity score. Reduced neutrophil infiltration into colon tissue was associated with a marked reduction in plasma CXCL1 levels. Of note, our results are in line with other mouse colitis models, showing that shifting the balance of anticoagulant pathways to a procoagulant state aggravates colitis (Vetrano et al., 2011). The correlation of an over-activation of the coagulation system in active IBD is supported by a wealth of clinical data (Lee et al., 1968; Boehme et al., 1997; Saibeni et al., 2010; Deutschmann et al., 2013; Schmid et al., 2014). Importantly our study, applying the DSS-model of acute intestinal inflammation on *F9*-deficient hemophilia B mice, causally demonstrated that the FIX-dependent formation of FXa *in situ* is most likely one of the factors that exacerbates the symptoms of colitis. In conclusion, our findings support the extra circulation source of coagulation factor IX and its expression is increased in intestinal epithelial cells upon TLR stimulation. So far little is known about the effects of direct oral anticoagulants on the symptoms of active IBD. Future studies should focus on the role of ectopic synthesis of coagulation factors in active IBD and investigate the role of coagulation factor signaling in the recruitment of inflammatory cells, its influence on epithelial renewal and the regulation of intestinal barrier function.

## MATERIALS AND METHODS

### Mice

All mice were housed in a barrier facility (TARC, Translational Animal Research Center, University Medical Center Mainz) under specific pathogen-free (SPF) conditions with a 12 h light–dark cycle. They were kept in EU Type II IVC cages with 2–5 mice per cage and given standard lab diet (PMI LabDiet 5021, St. Louis, USA) and water *ad libitum*, in a 22°C±2°C room. *F9^−/−^* mice (Lin et al., 1997) were originally purchased from The Jackson Laboratory (Bar Harbor, USA) and the colony was maintained by hemizygous breeding. Hence, the C57BL/6J WT control mice (*F9^+/+^*) analyzed in this study were kept in the same cages as *F9^−/−^* mice also after weaning. All mice used in the experiments were 8–16-week-old male and female mice. All groups of mice were co-housed, and sex-, age- and weight- matched. All mice used for experiments were free of clinical symptoms. All procedures performed on mice were approved by the Animal Care and Use Committee (ACUC; Landesuntersuchungsamt Rheinland-Pfalz, Koblenz, Germany; 23 177-07/G 12-1-035).

### DSS-induced colitis model and tissue preparation

Acute colitis was induced by providing 3.5% DSS solution (MW 36–50,000; MP Biomedicals, Solon, USA) dissolved in autoclaved water for 7 days, with daily consumption monitored for any strain specific reluctance. The mice were weighed and randomized into four groups. A normal control group comprising WT (*n*=5), *F9^−/−^* (*n*=9) received autoclaved water without DSS orally for 7 days. A colitis group comprising WT (*n*=6) or *F9^−/−^* (*n*=8) received 3.5% DSS in autoclaved tap water orally for 7 days. Mice were checked daily for their disease activity index. On day 7 the animals were euthanized by cervical dislocation and organs including small intestine, liver and blood were harvested, processed and stored at −80°C for further analysis. Colons were excised upon autopsy and colon lengths were measured.

### Evaluation of colitis

To reflect the general condition of mice, a disease activity index was determined by an investigator, blinded to the protocol, by scoring the extent of body-weight loss, stool guaiac positivity or gross bleeding, and stool consistency (Table 1) according to the method of Murthy et al. (Murthy et al., 2002). Briefly, the mice were weighed before starting DSS administration, during DSS treatment and on the day when the mice were euthanized. Stool consistency and the degree of blood in stool were evaluated every day until the end of the experiment using Haemoccult (Beckman Coulter, Krefeld, Germany).

**Table 1.**
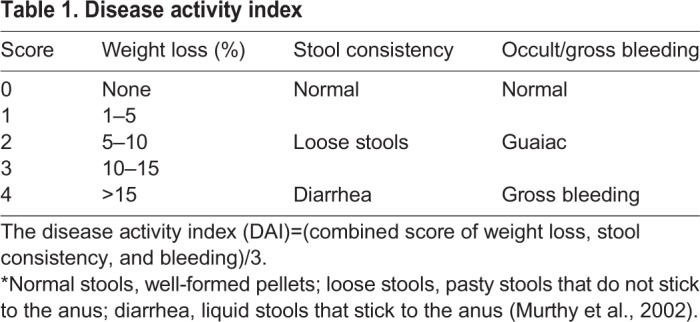
**Disease activity index**

### CXCL1 ELISA

CXCL1 concentrations were measured in murine plasma samples using an ELISA kit according to the manufacturer's protocol (R&D Systems, Minneapolis, USA).

### Myeloperoxidase activity assay

For myeloperoxidase (MPO) assay, organs were weighed, thoroughly washed in PBS and stored at −80°C until analysis. Plasma was collected and stored at −80°C, and was diluted in PBS before the analysis. Tissues were homogenized in in 0.5% hexadecyltrimethylammonium bromide (Sigma-Aldrich; H6269) in 50 mM PBS, pH 6.0. Homogenates were freeze-thawed thrice and centrifuged at 14,000 rpm at 4°C to remove the tissue debris. Supernatant and plasma was used for the MPO assay. The MPO assay was performed on clear supernatant and plasma in a 96-well plate by adding 1 mg/ml of dianisidine dihydrochloride (Sigma-Aldrich, D3252) and 0.00005% H_2_O_2_, optical density was measured at 450 nm. Human neutrophil MPO (Sigma-Aldrich, M6908) was used as a standard (range: 0.5-0.015 U/ml). One unit of MPO activity is defined as the amount needed to degrade 1.0 μmol of peroxide/min at 25°C.

### Mouse intestinal epithelial cells

The murine small intestinal epithelial MODE-K cell line was kindly provided by Dominique Kaiserlian (INSERM, Cedex, France) (Vidal et al., 1993). MODE-K cells were maintained as described. MODE-K cells were incubated with either vehicle or lipopolysaccharide (LPS, *Escherichia coli* 0111:B4; Sigma-Aldrich; 200 ng/ml), macrophage-activating lipopeptide-2 (MALP-2; Alexis, San Diego, USA; 2 μg/ml), Pam3CSK4 (InvivoGen, San Diego, USA; 200 ng/ml) for 2 and 4 h at 37°C. Cells were then harvested for further analysis.

### Immunoblotting

To extract the proteins, MODE-K cells or harvested small intestines were washed with PBS and homogenized in RIPA buffer containing 150 mM NaCl, 50 mM Tris-HCl (pH 7.4), 1 mM EDTA, 1% Triton X-100, 1% sodium deoxycholic acid, 0.1% SDS, 5 mM phenylmethylsulfonyl fluoride (PMSF), 10 µg/ml leupeptin and 20 µg/ml aprotinin with protease and phosphatase inhibitor cocktail (Roche, Basel, Switzerland). The crude extract was centrifuged at 10,000 ***g*** for 10 min at 4°C to remove cell debris. The supernatant was used as protein source and frozen at −80°C until further processing. The total protein was measured by Nanodrop (Thermo Fisher Scientific). The total protein extracts were loaded on 8–10% SDS-PAGE and separated by electrophoresis. The protein bands were transferred onto PVDF membranes, which were blocked and incubated overnight with primary antibodies; anti-factor IX (5 µg/ml, Acris, San Diego, USA) and anti-Actin (1:1000, Sigma-Aldrich). Membranes were washed and incubated with horseradish peroxidase-linked (HRP) secondary antibodies (1:3000, Santa Cruz Biotechnology). Proteins were visualized by enhanced chemiluminescence according to the manufacturer′s instructions (Cell Signaling Technology). For densitometric analysis of protein bands, the software ImageJ (NIH) was applied.

### Statistical analysis

All values are expressed as the mean±s.d. Data sets were analyzed with GraphPad Prism 6 (GraphPad) using one-way analysis of variance (ANOVA) and paired *t*-tests. Differences of *P*<0.05 were considered statistically significant.

## Supplementary Material

First Person interview
