# Supplementary material for: Coagulation factor 9-deficient mice are protected against dextran sulfate sodium-induced colitis
Source: Biol Open. 2018 Jun 26;7(7):bio034140. doi: 10.1242/bio.034140 (PMC6078354; doi:10.1242/bio.034140)
Supplement: First Person interview [file biolopen-7-034140-s1.pdf]

FIRST PERSON

First person – Avinash Khandagale

First Person is a series of interviews with the first authors of a selection of papers published in Biology Open, helping early-career researchers promote themselves alongside their papers. Avinash Khandagale is first author on 'Coagulation factor 9-deficient mice are protected against dextran sulfate sodium-induced colitis', published in BiO. Avinash conducted the research in this article while a postdoc in the lab of Christoph Reinhardt at the Centre for Thrombosis and Haemostasis, University Medical Centre, Mainz, Germany. He is now a senior researcher in Agneta Siegbahn's lab at Uppsala University Hospital, Uppsala, Sweden, investigating the role of extracellular vesicles in plasma produced by platelets and platelet function in heart disease.

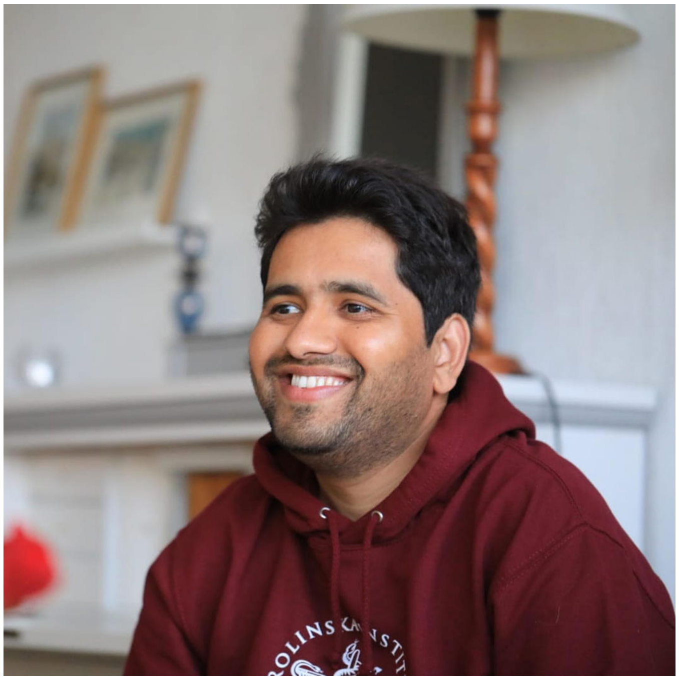

Avinash Khandagale

What is your scientific background and the general focus of your lab?

My academic training and research experience during my PhD in the lab of Prof. Bernd Engelmann at LMU, Munich, Germany, have provided an excellent background in multiple biological disciplines, including cell biology, thrombosis and haemostasis. My particular interest in preclinical research led me to move to Prof. Christoph Reinhardt's research group at the Centre for Thrombosis and Haemostasis, University Medical Centre, Mainz, Germany. The lab's research focuses on coagulation factor signalling in the small intestine, together with the identification of systemic effects that enhance thrombus formation in the context of the presence of intestinal microbiota. Germ-free mice are powerful tools to resolve the effect of commensal microbiota on the host organism. To this end, our group established germ-free mouse colonies to study the influence of microbiota on the host's coagulation system and on thrombus formation.

How would you explain the main findings of your paper to non-scientific family and friends?

Inflammatory bowel disease (IBD) is a group of inflammatory conditions of the large intestine (colon) and small intestine of the gastrointestinal tract, making the intestine red, swollen and painful. Patients with IBD are susceptible to the formation of blood clots in their veins, resulting in the blockage of oxygen and nutrient supplies to their vital organs. This development of blood clots in turn aggravates inflammation during IBD. Various blood coagulation factors are involved in the formation of clots. We, for the first time, identified possible local synthesis of blood coagulation factors (specifically, one known as coagulation factor IX) in the layer of cells that forms the internal surface of both the small intestine and the colon during inflammatory conditions. Imagine you are passing through a tunnel and see the inside walls of the tunnel are damaged due to leakage, and you find this damage is increased due to the growth of plant roots at the site of damage. Similarly, we found local production of coagulation factor IX, which resulted in increased inflammation (damage) to intestinal walls from the inside. We treated mice with a specific chemical through drinking water that resulted in inflammation of the intestine and found that mice with a deficiency of coagulation factor IX were protected from chemically induced intestinal damage. Moreover, it is known that individuals with haemophilia (with factor IX deficiency) are resistant to the development of IBD. This finding can potentially open up new therapeutic target exploration in IBD.

What are the potential implications of these results for your field of research?

Our paper presents a novel experimental finding of a reduced colitis phenotype in coagulation factor IX-deficient mice. So far, little is known about the effects of direct oral anticoagulants on the symptoms of active IBD. The findings of the present study provide compelling evidence that factor IX is locally produced in the intestinal epithelial cells and its expression increases upon stimulation with microbial products. Moreover, the disease phenotype is significantly reduced in factor IX-deficient mice in experimental IBD. From a clinical perspective, it is increasingly recognized that therapeutic approaches based on direct coagulation factor inhibition, rather than traditional multi-targeted anticoagulants (e.g., warfarin and unfractionated heparin), are likely associated with a wider therapeutic window. We herein provide proof of principle that targeting coagulation factor IX locally in the intestine during the propagation of IBD may be therapeutically beneficial.

What has surprised you the most while conducting your research?

Our observation of the expression of factor IX in murine small intestine as well as an intestinal epithelial cell line was very surprising because of the fact that so far, in our understanding, the liver is known

Avinash Khandagale's contact details: Department of Medical Sciences, Coagulation and Inflammation Sciences, Academic Hospital, 751 85 Uppsala, Sweden.

E-mail: Avinash.Khandagale@medsci.uu.se

Biology Open (2018): doi:10.1242/bio.034140: Supplementary information

to be the primary source of F9 synthesis. This observation strengthens the notion of extra-hepatic expression of various coagulation factors. The second surprising and rather interesting observation was to confirm the epidemiological finding that haemophilia B provides resistance to IBD in a murine model of haemophilia B.

What, in your opinion, are some of the greatest achievements in your field and how has this influenced your research?

Increased use of gnotobiotic mouse models in biological research has improved our understanding of the role of the endogenous microbiota in host development and disease progression. However, use of gnotobiotics in thrombosis research and related disease conditions is still under-utilized. Inflammatory conditions during bowel disease are characterized by a dysbiotic gut microbiota and it is now well known that gut microbes control the development of IBD. Interestingly, encouraging results are currently being published from the first clinical trials to test faecal microbiota transplantation (FMT) as a new therapeutic option in IBD.

"Unless we are outstanding in the science of today, it will not be possible to answer the needs of the technology of the future."

What changes do you think could improve the professional lives of early-career scientists?

I am an advocate of cutting-edge research because I believe today's science is tomorrow's technology. Unless we are outstanding in the science of today, it will not be possible to answer the needs of the technology of the future. It is therefore essential that we invest more in basic research to understand fundamental biological processes to tackle the technology of tomorrow. However, as a basic biology researcher, it is getting increasingly difficult to attract funds. One of the major problems for early-career scientists is a lack of dedicated funding possibilities. It is very difficult for early-career researchers, and especially those in developing countries, to compete with established professors. Support and guidance from both mentors and institute administration is crucial to improve the professional development of early-career scientists.

What's next for you?

I have enjoyed doing research over the last 10 years. It fascinates me to work and experiment with new things every time I hit my bench. I wish to continue learning and being surprised with new aspects of human biology for a long time to come. Soon I will be starting a Forskare (senior researcher) position in translational cardiovascular research at Uppsala University, Sweden, to identify biomarkers in disease conditions. I am currently exploring early-career faculty positions, where I can use the skills that I have developed during postdoctoral training, and continue research on disease modelling and biomarker identification to help contribute to the betterment of a healthy society.

Reference

Khandagale, A., Kittner, J. M., Mann, A., Ascher, S., Kollar, B. and Reinhardt, C. (2018). Coagulation factor 9-deficient mice are protected against dextran sulfate sodium-induced colitis. *Biol. Open* **7**: bio034140.
